# Supplementary material for: Visual processing mode switching regulated by VIP cells
Source: Sci Rep. 2017 May 12;7:1843. doi: 10.1038/s41598-017-01830-0 (PMC5432022; doi:10.1038/s41598-017-01830-0)
Supplement: Supplementary file 1 — Visual processing mode switching regulated by VIP cells [file 41598_2017_1830_MOESM1_ESM.pdf]

Visual processing mode switching regulated by VIP cells

Jung Hoon Lee[1*] and Stefan Mihalas[1]

[1]Allen Institute for Brain Science

jungl@alleninstitute.org

Supplementary Figures

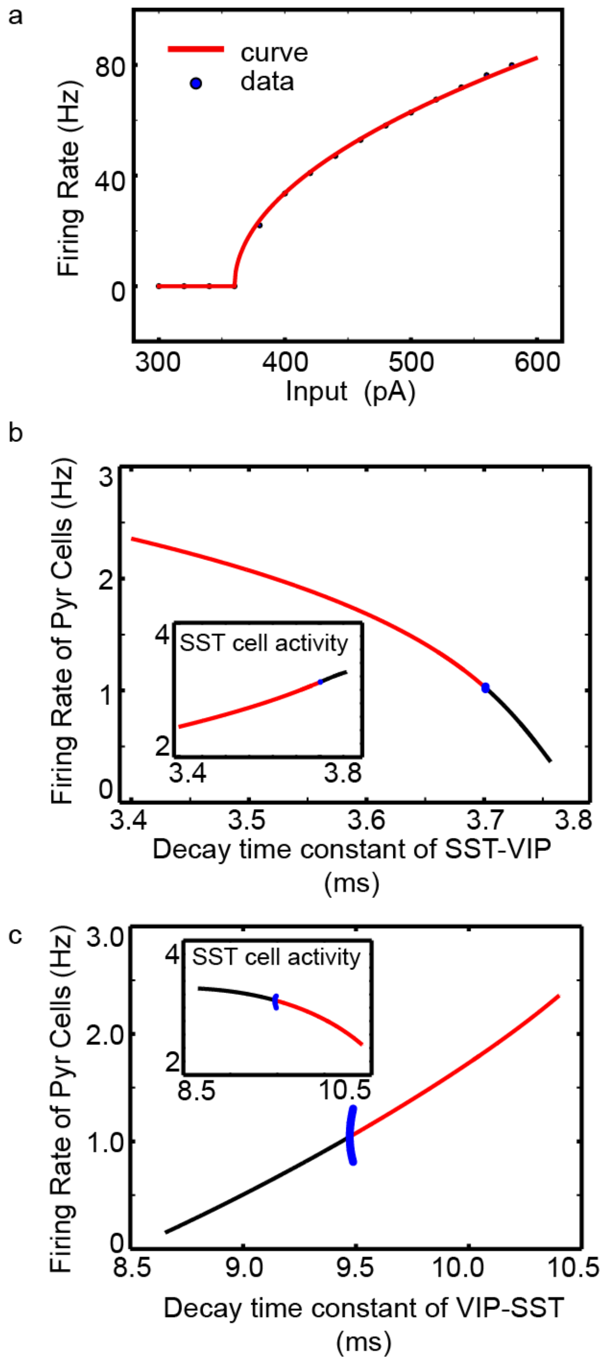

Supplementary Figure 1: **Local circuit properties. (a)**, The F-I curve of a leaky integrate and fire neuron specified in supplementary Table 1. The red curve shows the gain function ($5.33\sqrt{x-360}$) of our firing rate model. **(b)** and **(c)**, bifurcation analyses. In this study, the external inputs to Pyr, PV, SST and VIP cells are 3.5, 4.0, 1.0 and 0.6 pA, respectively. The bifurcation parameters are the decay time constants of SST-VIP in (b) and VIP-SST connections in (c). The insets in (b) and (c) show the SST cell activity in the same analyses.

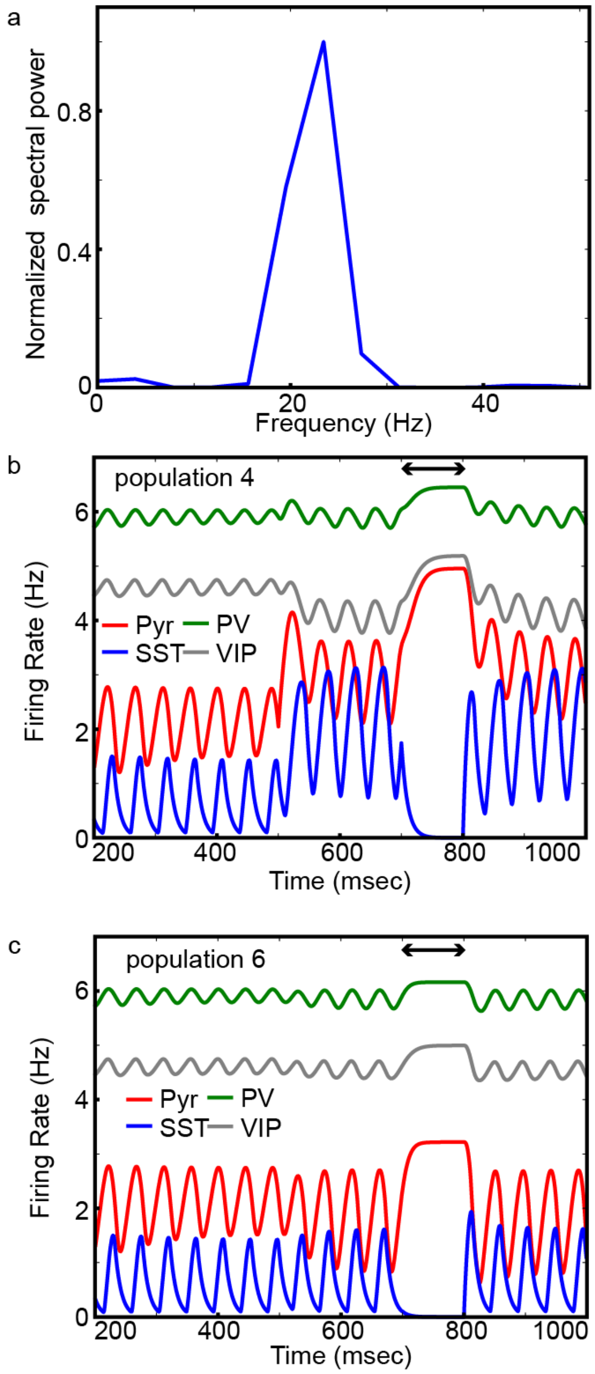

Supplementary Figure 2: Oscillatory responses. (a), Spectral power of Pyr cell output, which is normalized to the maximum value. **(b)** and **(c)**, Responses of populations 4 and 6 with the inhibitory current to SST cells between 700-800 ms (marked with the black arrow).

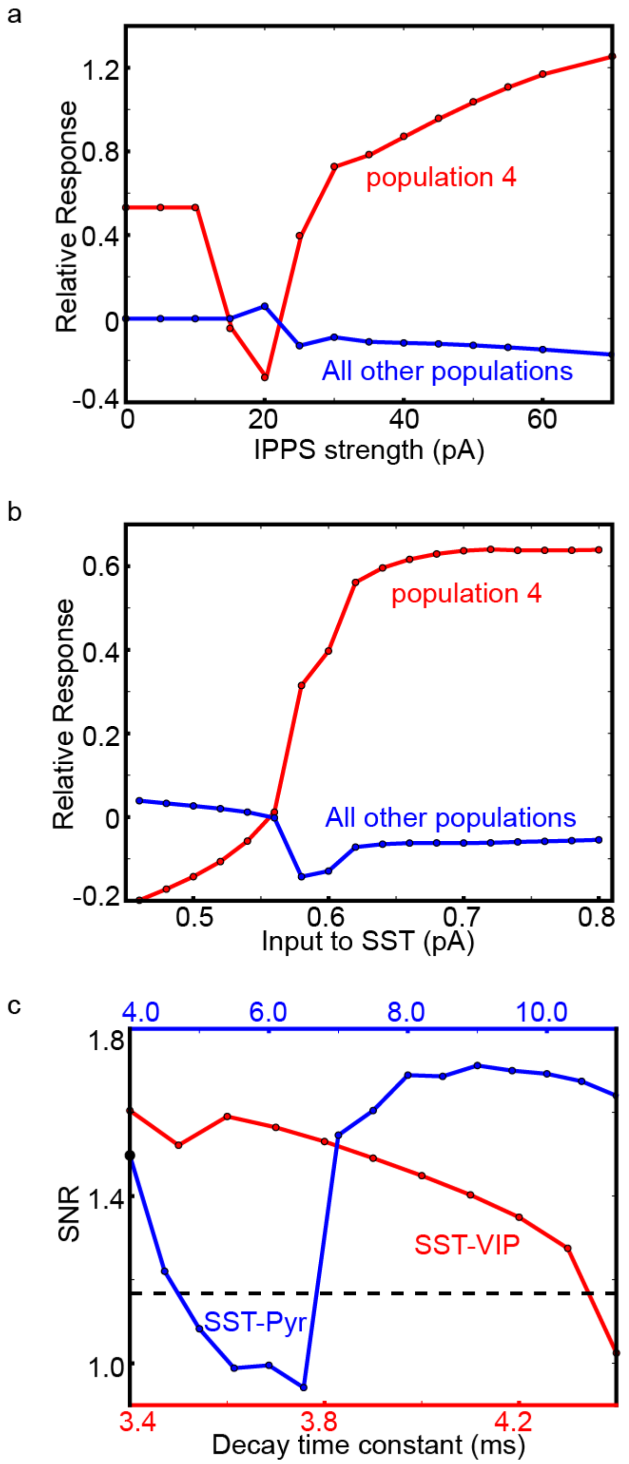

Supplementary Figure 3: The dependency of model response. **(a)**, Stimulus-period Pyr cell activity in relative to the baseline activity (see Methods) depending on the IPPS strength. The red and blue lines show population 4 and the mean activity of all others. **(b)**, the same as (a) but as a function of the input to SST cells. **(c)**, SNR depending on the decay time constants of Pyr-SST and SST-VIP connections.

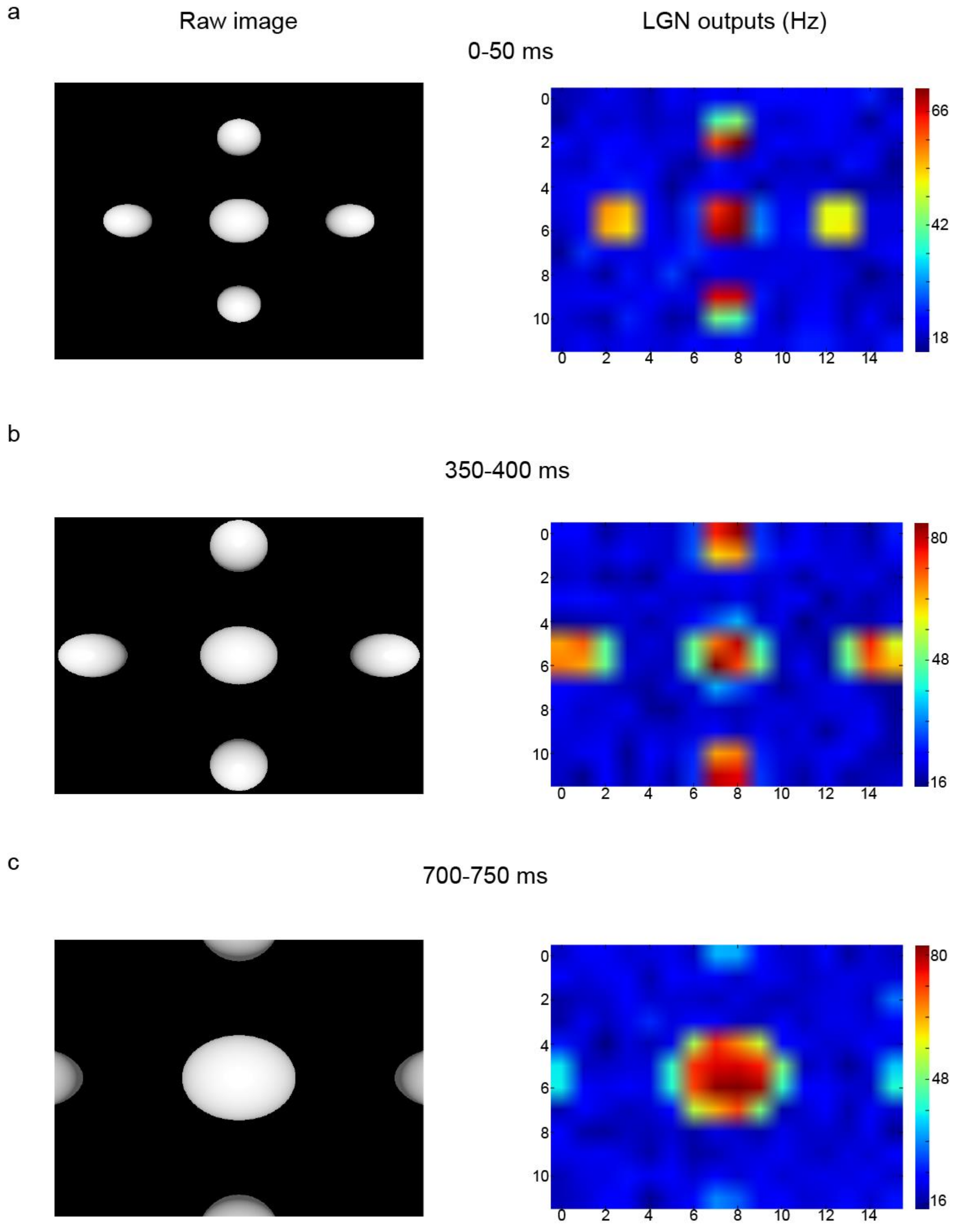

Supplemental Figure 4: The raw images and LGN outputs. The left and right columns show rendered images during 1 sec simulation translation at a constant speed toward the screen (the simulated retinal image) and corresponding LGN firing rates, respectively. Each 16 by 12 grid excites 100 LGN cells whose average firing rates are shown here.

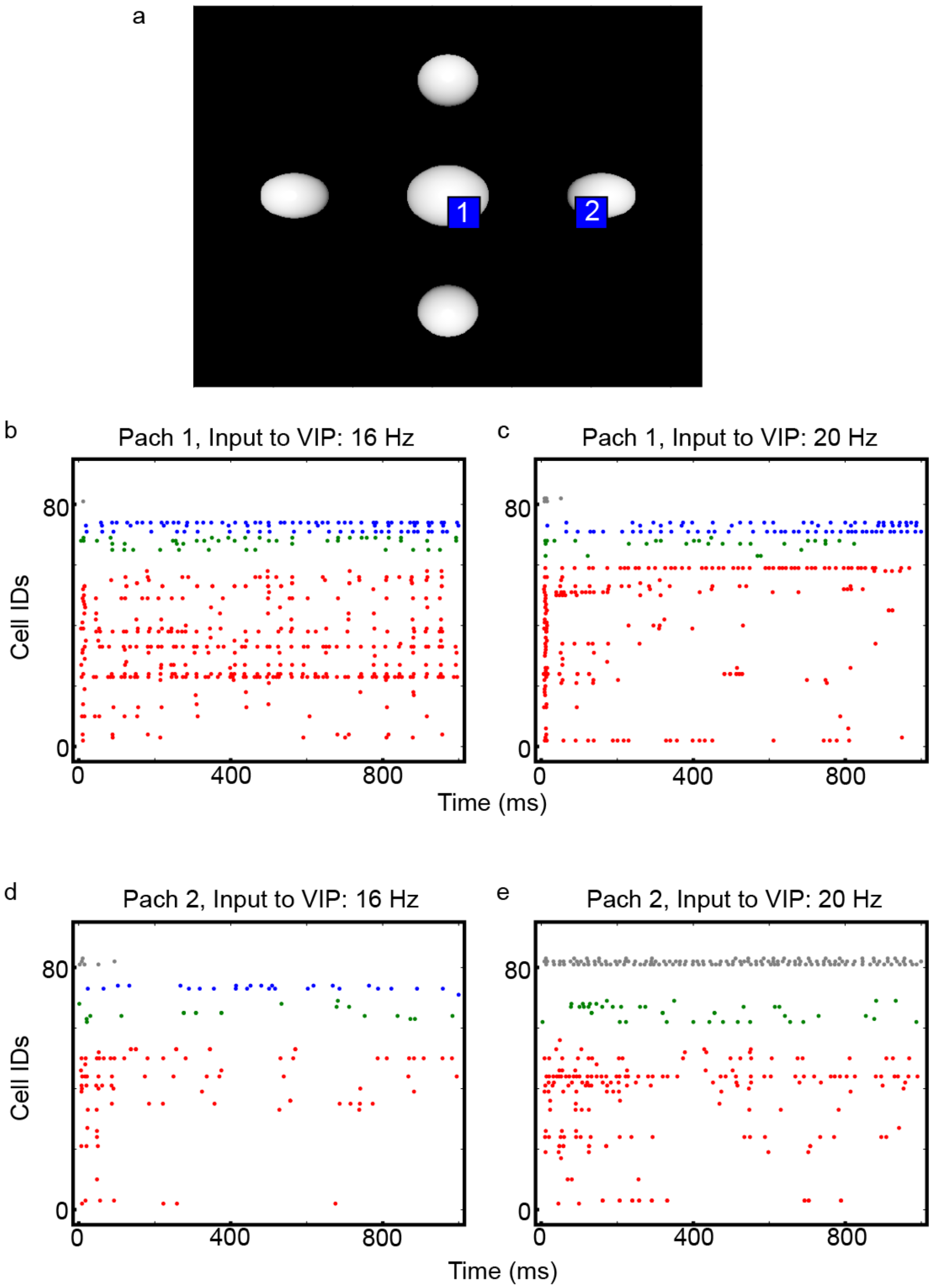

Supplemental Figure 5: Examples of cell type response. (a), Location of two image patches. **(b)** and **(c)**, Cell responses to the patch 1 in the high and low depolarization conditions, respectively. Red, green, blue and gray dots are action potentials of Pyr, PV, SST and VIP cells, respectively. Each row shows action potentials of an individual cell; cell ids are nominal. The empty rows represent inactive cells, and we add additional space between cell types for the clarity. **(d)** and **(e)**, cell responses to patch 2.

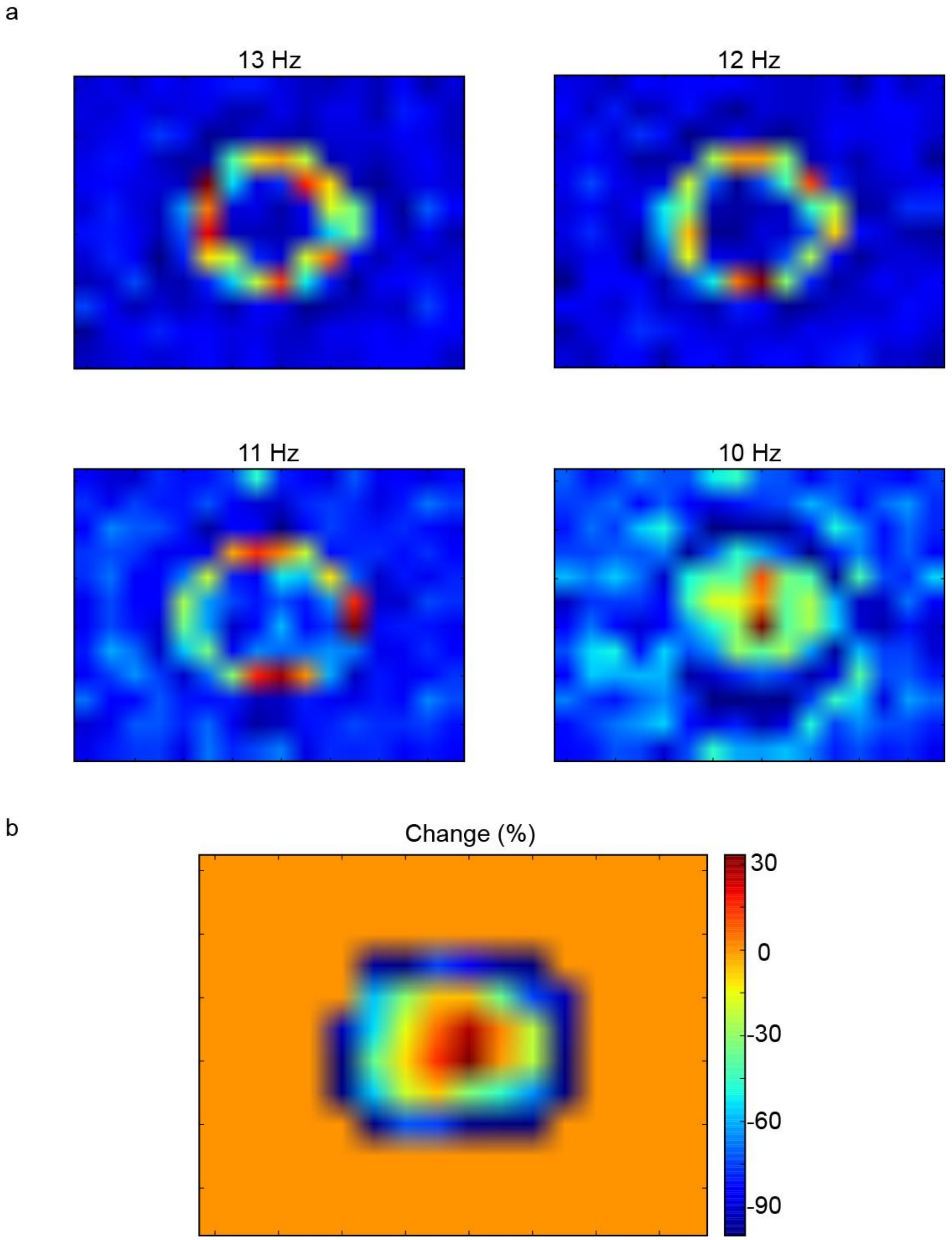

Supplementary Figure 6: The dependency of column responses on PV cells' excitability. (a), Averaged column responses between 950-1000 ms from 10 independent simulations. We display them depending on different background inputs to PV cells. The input strengths are shown above each panel. **(b)**, Modulation of SST cell activity induced by VIP cell depolarization in the last frame 950-1000 ms. We calculate the relative increase. The red represents 30% increase, and 0% change represents the areas where SST cells are quiescent even with low VIP cell activity.

Supplementary Tables

Supplementary Table 1: Parameters for synaptic connections. This table lists the default values used unless stated otherwise.

Synaptic connections	T (ms)	Within population (pA)	Across population (pA)
Pyr-SST	2.0	80	25
Pyr-PV	2.0	80	N/A
Pyr-VIP	2.0	20	N/A
Pyr-Pyr	2.0	40	5
PV-PV	4.3	-120	N/A
PV-Pyr	6.0	-80	N/A
SST-Pyr	7.5	-40	N/A
SST-PV	3.4	-30	N/A
SST-VIP	3.4	-40	N/A
VIP-SST	10.4	-35	N/A

Supplementary Table 2: Intrinsic input to cell types. The intrinsic inputs are the sum of spiking thresholds and external background inputs. As the spiking threshold (-375 pA) is identical among all cell types, the intrinsic inputs shown below correspond to the strength of background input.

Cell types	$I_{intrinsic}$ (pA)
Pyr	3.0
PV	4.0
SST	0.6
VIP	0.75

Supplementary Table 3: Parameters used in the computational model. This table shows the parameters selected for the two-dimensional model consisting of 16-by-12 columns. The cell-type specific lateral connections are established when the distance between the two columns is shorter than the lengths specified below. In our simulations, all cells receive background external inputs via external fibers, each of which carries Poisson spike trains, as in the earlier model[27]. The cell-type specific fiber number and the frequency of Poisson inputs are given below. The connection probabilities are taken from the earlier models[12,15].

Peak currents (pA), $w \pm \delta w$	
default excitatory	175.6 ± 17.6
default inhibitory	-702.4 ± 70.2
PV→Pyr	-466.7 ± 46.7
PV→PV	-638.1 ± 63.8
PV→VIP	-140.0 ± 14.0
SST→Pyr	-200.0 ± 20.0
SST→PV	-228.6 ± 22.9
SST→VIP	-525.8 ± 52.6
VIP→Pyr	-76.2 ± 7.62
VIP→SST	-66.7 ± 6.7
L4E→Pyr	245.8 ± 24.6
Thalamus→Cortex	1580.4 ± 158.0

Decay time constants (ms)	
Pyr→Pyr	2.0
PV→Pyr	6.0
SST→Pyr	7.5
VIP→Pyr	6.2
Pyr→PV	2.0
PV→PV	4.3
SST→PV	3.4
Pyr→SST	2.0
VIP→SST	10.4
Pyr→VIP	2.0
PV→VIP	4.3
SST→VIP	3.4
default exc	0.5
default inh	0.5

Neuron Params
τ: 10 ms, V_{th}: -50 mV, V_{reset}: -65 mV, τ_{ref}: $3\ ms$, C: 250 pF

Neuron # in a single column	
L2/3	E:517 I:146
L4	E:548 I:137
L5	E:121 I:27
L6	E: 360 I: 74

Synaptic delay (ms), $d \pm \delta d$	
Intracolumnar exc.	1.5 ± 0.75
Intracolumnar inh.	0.75 ± 0.375
Intercolumnar exc.	7.5 ± 3.75
Intercolumnar inh.	3.75 ± 1.88

Connection Probabilities for intercolumnar connections		
Connections	Conn. probability (%)	Length of lateral conn.
Pyr-SST	1.5	4
Pyr-PV	4.0	2
PV-Pyr	5.0	1
Pyr-Pyr	8.0	1

Background inputs	
Layer	External fiber #
L2/3	Pyr: 1600 Inh: 1500
L4	Exc: 2100 Inh: 1900
L5	Exc: 2000 Inh: 1900
L6	Exc: 2900 Inh: 2100

Background spike rate (Hz) per external fiber	
L2/3	Pyr: 9.0 PV:12.0 SST:1.2 VIP: 16.0/20.0
All other layers	Exc: 8.0 Inh:8.0

Peak currents $w \pm \delta w$ of background inputs
$44.0 \pm 4.4\ pA$
